# Supplementary material for: Close Cardiovascular Monitoring during the Early Stages of Treatment for Patients Receiving Immune Checkpoint Inhibitors
Source: Pharmaceuticals (Basel). 2024 Jul 21;17(7):965. doi: 10.3390/ph17070965 (PMC11279915; doi:10.3390/ph17070965)
Supplement: Supplementary file 1 [file pharmaceuticals-17-00965-s001.zip › pharmaceuticals-3096694-supplementary.pdf]

Supplementary

Table S1. Three-dimensional echocardiography parameters at baseline and three months for the subgroup of patients who had coronary artery disease, i.e., 10/54.

Parameters	*n*	Baseline	3 months	*p*
3D-LVEF (%)	7	50 [43 ; 58]	49 [43 ; 64]	0.50
LVEDV (mL)	7	132 [110 ; 169]	135 [102 ; 170]	0.23
LVESV (mL)	7	68 [50 ; 87]	81 [40 ; 98]	0.18
GLS (%)	7	-14.1 [-15.7 ; -8.8]	-14.9 [-15.4 ; -11.9]	0.080
2-chamber (%)	7	-12.3 [-15.0 ; -10.0]	-14.10 [-16.5 ; -11.7]	0.46
3-chamber (%)	7	-14.1 [-18.9 ; -8.7]	-15.9 [-18.0 ; -12.1]	0.18
4-chamber (%)	7	-12.8 [-15.8 ; -10.0]	-10.9 [-15.7 ; -10.0]	0.74
Right ventricular function				
TAPSE (mm)	8	22 [20 ; 25]	21 [16 ; 25]	0.89
s'-wave (cm/s)	9	11 [9 ; 13]	12 [8 ; 13]	0.40
Dimensions				
Left atrial area (cm^2)	9	19 [16 ; 22]	21 [15 ; 23]	0.16
Diastolic function				
E (cm/s)	9	50 [47 ; 71]	55 [46 ; 99]	0.085
A (cm/s)	9	70 [61 ; 97]	77 [64 ; 89]	0.869
E/A ratio	9	0.7 [0.6 ; 0.9]	0.7 [0.5 ; 1.6]	0.34
Deceleration time (ms)	9	177 [131 ; 238]	226 [148 ; 232]	0.59
Peak e´ velocity of septal wall (cm/s)	9	6 [5 ; 8]	6 [5 ; 8]	1.00
Peak e´ velocity of lateral wall (cm/s)	9	7 [6 ; 8]	9 [7 ; 10]	0.23
E/e´ septal wall	9	9 [9 ; 12]	13 [7 ; 15]	0.26
E/e´ lateral wall	9	8 [6 ; 11]	8 [5 ; 13]	0.95
MV E/e´ average	9	8 [7 ; 11]	7 [6 ; 13]	0.86

GLS: global longitudinal strain; LVEDV: left ventricular end-diastolic volume; LVEF: left ventricular ejection fraction; LVESV: left ventricular end-systolic volume; MV: mitral valve; TAPSE: tricuspid annular plane systolic excursion. n = number of patients. Values are median [IQR_1 ; IQR_3].

Table S2. Technical aspects of the different assays used to determine troponin T and I of the biobank samples at baseline and three months.

	Assay	n/N	Range of detection (ng/L)	Limit of blank (ng/L)	Limit of quantitation 10% (ng/L)	99th percentile normal cut-off (ng/L)
Hs-TnT	Elecsys e 801 (Roche Diagnostics)	108/108	3 – 10,000	2.5	13	14.0
Hs-TnI	Atellica IM (Siemens Healthineers)	108/108	2.5 – 25,000	0.5	6	45.2 (male 53.5; female 34.1)

Table S3. Technical aspects of the different assays used to determine NT-proBNP of the biobank samples at baseline and three months.

Assay	n/N	Range of detection (pg/mL)	Limit of blank (pg/mL)	Limit of quantitation (pg/mL)	99th percentile normal cut-off (pg/mL)
Atellica IM (Siemens Healthineers)	52/108	35 – 35,000	20	35	125
Elecsys e 801 (Roche diagnostics)	56/108	5 – 35,000	3	50	125

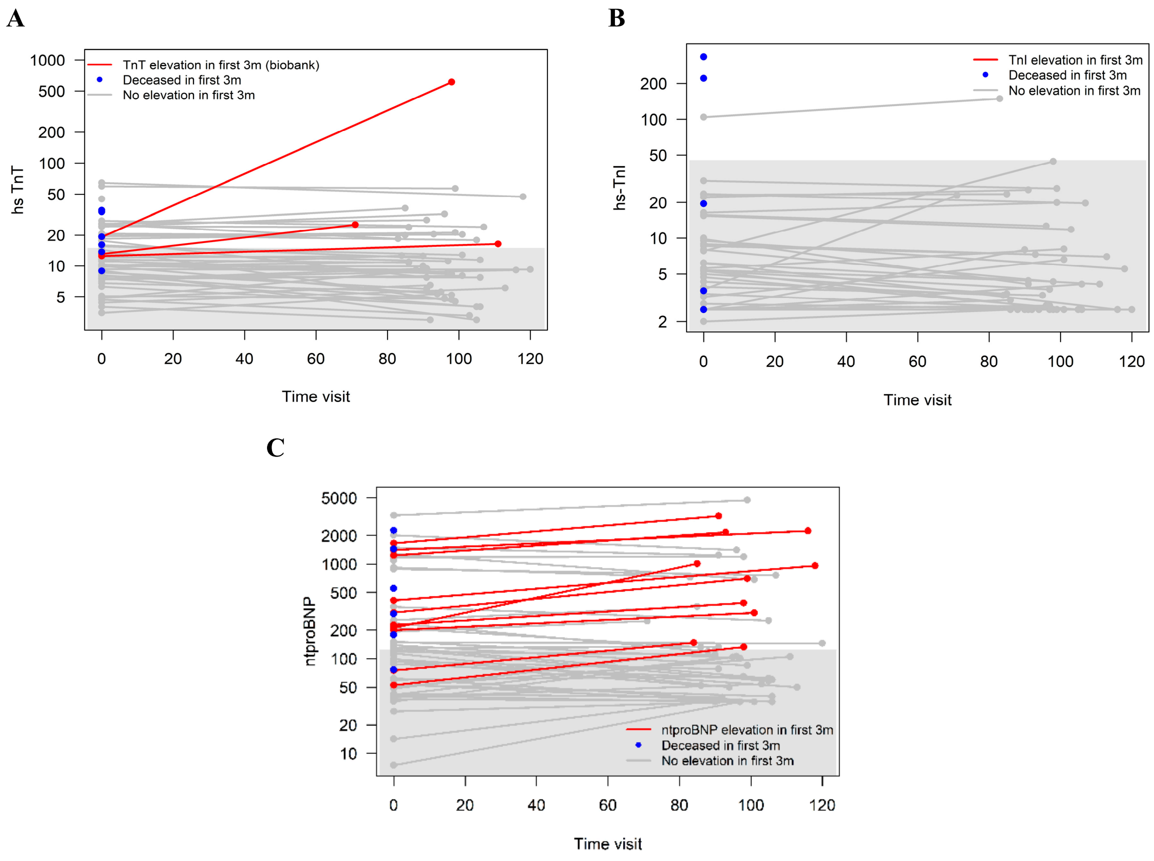

Figure S1 Evolution of hs-TnT (A), hs-TnI (B) and NT-proBNP (C), measured at baseline and 3 months. Elevation was defined as follows: above the upper limit of normal (ULN) if the baseline value was normal; or 1.5 ≥ times baseline if the baseline value was above the ULN within the first three months of treatment. The gray zone in each graph represents the ULN.

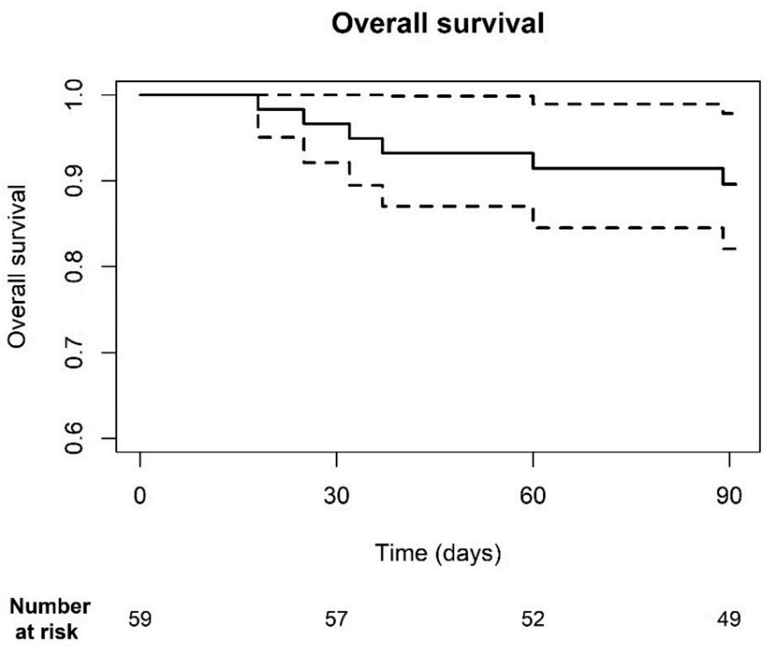

Figure S2 Overall survival up until 90 days after inclusion. Six patients died prior to their 3-month cardiology follow-up visit due to progressive disease. Four patients were followed-up for less than 90 days. The dotted line represents the 95% CI [82.0 – 97.8].
